# Comparative analyses of *Campylobacter concisus*strains reveal the genome of the reference strain BAA-1457 is not representative of the species

**DOI:** 10.1186/1757-4749-3-15

**Published:** 2011-10-13

**Authors:** Nadeem O Kaakoush, Nandan P Deshpande, Marc R Wilkins, Mark J Raftery, Karolina Janitz, Hazel Mitchell

**Affiliations:** 1 School of Biotechnology and Biomolecular Sciences, The University of New South Wales, Sydney, NSW 2052, Australia; 2 Systems Biology Initiative, School of Biotechnology and Biomolecular Sciences, The University of New South Wales, Sydney, NSW 2052, Australia; 3 Ramaciotti Centre for Gene Function Analysis, The University of New South Wales, Sydney, NSW 2052, Australia; 4 Biological Mass Spectrometry Facility, The University of New South Wales, Sydney, NSW 2052, Australia

**Keywords:** *Campylobacter concisus*, comparative, glycosylation, pseudoaminic acid, legionaminic acid

## Abstract

**Background:**

Several studies have shown that significant genotypic heterogeneity exists among *Campylobacter concisus *strains. Recently, the genome of *C. concisus *UNSWCD, isolated from a patient with Crohn's disease, was sequenced.

**Results:**

In this study, comparative analyses were performed between strain UNSWCD and BAA-1457, isolated from a patient with acute gastroenteritis. Searches between *C. concisus *UNSWCD and BAA-1457 showed that 76% of genes were homologues, whereas those between *C. jejuni *strains showed 90-91% to be homologues, indicating substantial variation exists within these two *C. concisus *genomes. More specific bidirectional homology searches identified 1593 genes that are shared between these strains, and 115 and 281 genes unique to UNSWCD and BAA-1457, respectively. Significantly, differences in the type of flagellin glycosylation pathways between the two strains were identified and confirmed by PCR. The protein profiles of UNSWCD, BAA-1457 and a further six strains of *C. concisus *were compared and analyzed bioinformatically, and this differentiated the strains into four clades. BAA-1457 was found to be highly divergent (average similarity: 56.8%) from the other seven strains (mean average similarity ± standard deviation: 64.7 ± 1.7%). Furthermore, searches for homologues of the 1593 proteins found to be common between UNSWCD and BAA-1457 were conducted against all available bacterial genomes, and 18 proteins were found to be unique to *C. concisus*, of which 6 were predicted to be secreted, and may represent good markers for detection of this species.

**Conclusions:**

This study has elucidated several features that may be responsible for the heterogeneity that exists among *C. concisus *strains, and has determined that the strain BAA-1457 is genetically atypical to other *C. concisus *strains and is not a good candidate reference strain.

## Background

*Campylobacter concisus *is a motile Gram-negative, spiral/curved, bacterium that requires a microaerobic hydrogen-enriched environment for growth [1]. Due to its association with acute enteritis and Crohn's disease (CD) [2-11], *C. concisus *has been described as an emergent pathogen of the human intestinal tract. However, the isolation of *C. concisus *from healthy individuals, and the failure of some studies to show a significant difference in the prevalence of *C. concisus *in subjects with diarrhea and healthy controls [4,5], has resulted in controversy regarding the role of *C. concisus *in intestinal disease.

Given that *C. concisus *has been reported to be genetically and taxonomically diverse with up to four or more genomospecies being described [3,12,13], we recently investigated the ability of a range of *C. concisus *strains to attach to and invade human intestinal epithelial cell lines using scanning electron microscopy (SEM) [14]. In that study, the adherence and invasive abilities of *C. concisus *UNSWCD, isolated from a CD patient, were compared with that of *C. concisus *strains UNSWCS and ATCC 51562 isolated from patients with acute gastroenteritis, and ATCC 51561 isolated from a healthy control. Based on the SEM results, *C. concisus *UNSWCD attached to and appeared to invade host cells [14]. While *C. concisus *strains from acute gastroenteritis or a healthy control also displayed flagellum-mediated attachment via microvilli, these strains did not appear to invade [14]. Based on these findings, Man *et al *quantified the invasive ability of the four *C. concisus *strains using gentamicin protection assays, and showed that the percentage invasion of *C. concisus *UNSWCD was more than 46- and 200-fold higher than that of *C. concisus *UNSWCS and *C. concisus *ATCC 51562, respectively [14]. Interestingly, *C. concisus *ATCC 51561 isolated from a healthy subject, showed no evidence of invasion.

Man *et al *further investigated the invasion process of *C. concisus *UNSWCD and found that host microtubules and microfilaments were involved due to the attenuation of invasion by inhibitors such as colchicine and cytochlasin D [14]. Interestingly, there is some consensus that *Campylobacter jejuni *strains may also require both microtubules and microfilaments during the invasion process into different intestinal cell lines [15-17], a finding that would suggest that *C. concisus *UNSWCD may use a similar mechanism of invasion to *C. jejuni*. Moreover, Man *et al *showed that *C. concisus *UNSWCD preferentially attached to intercellular junctional spaces and that this spatial distribution was concomitantly associated with a loss of membrane-associated ZO-1 and occludin [14].

As a result, Man *et al *postulated that the differences observed in the pathogenic potential of the *C. concisus *strains may be related to the genetic diversity among them [14]. To investigate this hypothesis, we recently sequenced the genome of *C. concisus *UNSWCD in order to compare it with the only other available *C. concisus *genome BAA-1457 (also known as strain 13826), a strain isolated from the feces of a patient with acute gastroenteritis [18]. A draft genome was assembled employing the *de novo *assemblers Velvet and Edena, and the UNSWCD genome size was found to be smaller (1.84 MB) when compared to its reference BAA-1457 counterpart (2.1 MB). Sequence comparisons to identify orthologues, essential gene verification analysis, syntenic association maps and proteomic validations by Orbitrap tandem mass spectrometry (more than 70% of the proteome identified), revealed a highly accurate assembly but one with significant differences to that of *C. concisus *BAA-1457. For example, clusters of syntenically placed genes in the *C. concisus *BAA-1457 genome were shown to be absent in the *C. concisus *UNSWCD genome, and thus, the functions associated with these genes are likely to be absent in UNSWCD [18].

In the current study, we analyzed the genomic and proteomic differences between *C. concisus *UNSWCD and BAA-1457 in order to examine the heterogeneity within this species and to identify putative factors responsible for the increased pathogenesis of *C. concisus *UNSWCD. Differences identified in the flagellin glycosylation of *C. concisus *UNSWCD and BAA-1457 were then assessed in a further six *C. concisus *strains.

## Results and Discussion

### Degree of diversity within Campylobacter concisus

Phylogenetic analyses of the 16S rRNA gene, internal transcribed region sequence and 23S rRNA gene of *C. concisus *UNSWCD and BAA-1457 have shown that these bacteria cluster together when compared to other *Campylobacter *species [19]. However, there remains high genetic diversity between the different *C. concisus *strains [3,12,13]. Studies have found that this species comprises several molecular groups (genomospecies). For example, Vandamme *et al *reported that fecal *C. concisus *isolates exhibited only 42 to 50% DNA-DNA hybridization values with strains of oral origin [13]. Further studies using pulsed field gel electrophoresis [12] and protein profiling [3] found *C. concisus *to comprise at least two genomospecies, which were phenotypically indistinguishable, but genetically divergent [12]. More recently, Aabenhus *et al *analyzed the genotype of 62 *C. concisus *clinical isolates using amplified length fragment polymorphism and showed that *C. concisus *contained at least four distinct genomospecies [2].

The recent sequencing of a second *C. concisus *genome (strain UNSWCD) [18], along with the available BAA-1457 genome provided us with the opportunity to perform comparative analyses of these genomes in an effort to identify differences among *C. concisus *strains. To understand the extent of variability between the genomes of the two *C. concisus *strains, bidirectional homology searches were performed and the values compared with that of searches between two pairs of the three *C. jejuni *strains with sequenced and comprehensively validated genomes. When cutoff values of 70% identity plus at least 85% gene length coverage were employed, searches between *C. concisus *UNSWCD and BAA-1457 showed that 76% of proteins were homologues, whereas those for *C. jejuni *NCTC 11168 and 81116 and NCTC 11168 and 81-176 showed 91% and 90% to be homologues, respectively. Calculation of the average percentage identities for all homologues revealed that for *C. concisus *UNSWCD and BAA-1457 it was 96% whereas that for the two *C. jejuni *pairs was 98%. Overall these values clearly indicate that even when compared to a phylogenetically related species, higher variation existed within the *C. concisus *genomes.

**Figure 1:**
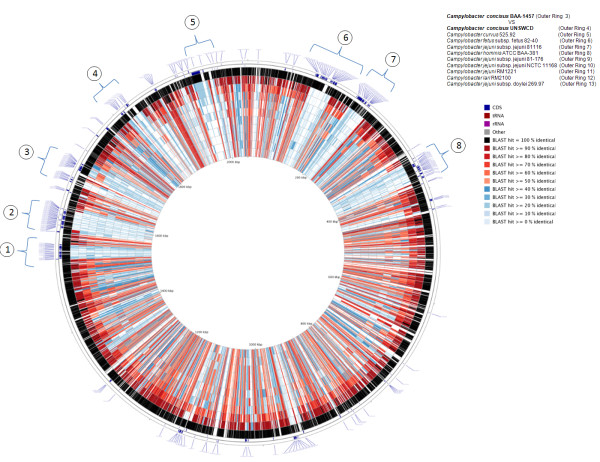
**Differences in gene content across the genomes of *C. concisus *BAA-1457, UNSWCD and other *Campylobacter *species**. Circular genome visualization was performed using the CGView webserver. **Ring I and II **(outer): coding sequences unique to *C. concisus *BAA-1457 when compared to other *Campylobacter *species; **ring III: ***C. concisus *BAA-1457; **ring IV: ***C. concisus *UNSWCD; **ring V: ***Campylobacter curvus *525.92; **ring VI: ***Campylobacter fetus *subsp. *fetus *82-40; **ring VII: ***Campylobacter jejuni *subsp. *jejuni *81116; **ring VIII: ***Campylobacter hominis *ATCC BAA-381; **ring IX: ***Campylobacter jejuni *subsp. *jejuni *81-176; **ring X: ***Campylobacter jejuni *subsp. *jejuni *NCTC 11168; **ring XI: ***Campylobacter jejuni *RM1221; **ring XII: ***Campylobacter lari *RM2100; and **ring XIII: ***Campylobacter jejuni *subsp. *doylei *269.97.

Further analyses of the differences in gene content across the genomes of *C. concisus *BAA-1457 and UNSWCD and other *Campylobacter *species were performed employing the CGView webserver (Figure 1). As expected, the genome with the highest homology to BAA-1457 was that of UNSWCD. Of interest were eight regions that were present in BAA-1457 yet were absent in all the other *Campylobacter *species including *C. concisus *UNSWCD (Figure 1). This provided initial evidence that the higher variation observed between the two *C. concisus *strains may be due to uncommon elements within the reference BAA-1457 strain.

More specific bidirectional homology searches for genes with greater than 40% similarity between the genomes of UNSWCD and BAA-1457 were performed. In addition, genes with 30-40% similarity were manually verified using functional domain and gene ontology analyses. This identified 1593 genes that are shared between these strains, representing a genome percentage similarity of 91.7% (1593/1738) for UNSWCD and 79.2% (1593/2010) for BAA-1457. The searches also identified a total of 115 (6.6%) and 281 (14.0%) genes unique to UNSWCD and BAA-1457, respectively (Additional file 1, Table S1; Additional file 2, Table S2), with a further 30 and 136 genes that were not categorized into either group due to insufficient information on these genes.

### Genomic variations unique to Campylobacter concisus BAA-1457

Of the 281 genes unique to BAA-1457 when compared to UNSWCD, 111 proteins they encoded were at a level of abundance within the cell that could be detected by mass spectrometric analyses [18]. The finding that these proteins were expressed when the bacterium was grown under normal conditions would indicate that they play an important role within the cell. The 281 genes encoded proteins with a variety of functions, including transport systems, energy metabolism and a secretion system, as well as 130 hypothetical proteins. Of specific interest was the identification of at least four proteins involved in a Type VI secretion apparatus, CCC13826_1177, CCC13826_1178, CCC13826_1182 and CCC13826_1188, all of which were expressed under normal conditions (Additional file 1, Table S1). Type VI secretion systems (T6SS) have been identified in many bacterial pathogens including *Vibrio cholerae, Pseudomonas aeruginosa, Yersinia pestis *and *Salmonella enterica*, and have been implicated in the virulence of all of these species [20]. Although T6SS are employed to transport proteins across the bacterial envelope, more recently, a family of T6SS proteins have been found to share structural features with the cell-puncturing device of the T4 bacteriophage, and thus, may be used by the bacterium to puncture host cell membranes and insert the T6SS apparatus into the host cytosol [21].

**Figure 2:**
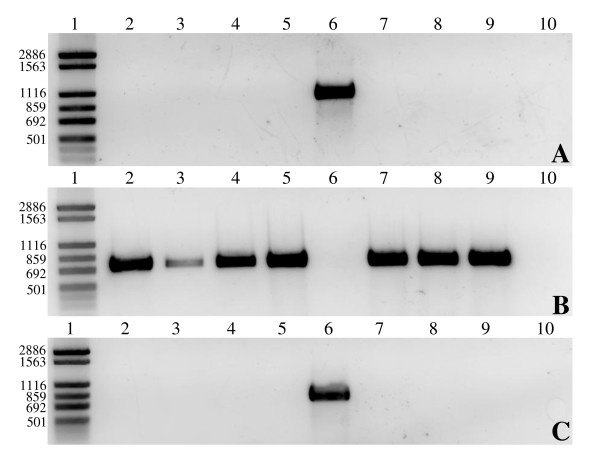
**PCR analyses of the *zot *(A), *pseB *(B) and *legB *(C) genes in the eight *Campylobacter concisus *strains**. Lane 1: FN-1 marker, lane 2: UNSWCD, lane 3: UNSW2, lane 4: UNSW3, lane 5: UNSW1, lane 6: BAA-1457, lane 7: UNSWCS, lane 8: ATCC 51562, lane 9: ATCC 51651 and lane 10: negative control.

Another protein of interest found within the unique proteins of BAA-1457 was the zonula occludens toxin (Zot). This protein and its possible functions within the cell have been previously discussed by Kaakoush *et al *[22]. Briefly, Zot is known to mimic a physiological modulator of intercellular tight junctions [23], and is used by virulent pathogens such as *Vibrio cholerae *and *Neisseria meningitidis *to increase tissue permeability [24]. The presence of Zot in BAA-1457 and its absence in UNSWCD was confirmed by PCR (Figure 2A). The presence of this gene in a further six strains of *C. concisus *(Table 1) was also investigated, and the toxin was found to be absent in all six strains (Figure 2A) (results were confirmed with the second primer pair ZotF/Zot2), suggesting that it is an uncommon feature within the genomes of *C. concisus *strains.

**Table 1:** *Campylobacter concisus *strains used in this study.

Strain	Source of isolation	Disease	Reference
UNSWCD	Human intestinal biopsy	Crohn's disease	[11]
UNSW1	Human intestinal biopsy	Chronic gastroenteritis	[Kaakoush *et al*, unpublished data]
UNSW2	Human intestinal biopsy	Crohn's disease	[Kaakoush *et al*, unpublished data]
UNSW3	Human intestinal biopsy	Crohn's disease	[Kaakoush *et al*, unpublished data]
ATCC 51561	Human feces	Healthy	[13]
ATCC 51562	Human feces	Acute gastroenteritis	[13]
UNSWCS	Human feces	Acute gastroenteritis	[14]
BAA-1457	Human feces	Acute gastroenteritis	[41]

### Genomic variations unique to Campylobacter concisusUNSWCD

Due to the isolation of UNSWCD from a patient with CD and its increased invasive potential as compared with other *C. concisus *strains [14], the unique features within this strain compared to BAA-1457 were of particular interest. The 115 genes unique to UNSWCD encoded 37 proteins that were at a level of abundance within the cell that could be detected by mass spectrometric analyses (Additional file 2, Table S2) [18]. They comprised proteins with functions including transport, oxidative stress and carbohydrate metabolism, as well as 88 hypothetical proteins. For example, a heat shock protein G homologue found to be involved in protection against oxidative stress in several bacterial species including Cyanobacteria was identified [25].

Several proteins involved in phage protection were also identified, including a phage portal protein, and a phage antirepressor protein. In addition, a modification methylase and endonuclease were identified. Modification methylases are involved in restriction-modification systems that are responsible for producing a species-characteristic methylation pattern in a short sequence in the host cell's DNA. Thus, any DNA from another species that gains entry into the cell and lacks the characteristic methylation pattern are recognized by restriction endonucleases and destroyed by cleavage. In addition, proteins involved in the processing of exogenous DNA and DNA repair were found, namely a competence protein CoiA and a nucleotidyltransferase family protein. While there are limited studies on the function of CoiA, Desai and Morrison have reported that this protein although not involved in uptake of donor DNA, is involved in processing this DNA to make viable mutants [26]. The presence of these proteins in UNSWCD may suggest that this strain has a more adaptable nature that may have lead to its increased efficiency in the host.

A further protein, an O-antigen ligase, that may play a role in the aggregation and adherence of *C. concisus *UNSWCD onto host cells, was also identified. This protein functions in the attachment of polymerized O-antigen repeat units to the lipid A core, and has been found to be important for cell wall integrity and bacterial motility [27]. More recently, Morgenstein *et al *found that deactivation of the O-antigen ligase in *Proteus mirabilis *had little effect on its swimming motility in soft agar but blocked its swarming motility on solid surfaces [27]. Thus, this protein may aid *C. concisus *UNSWCD to aggregate on host cells as has been observed previously by SEM [14].

Another protein of interest that was unique to *C. concisus *UNSWCD when compared to BAA-1457, and to other members of the Campylobacterales, was the Acr protein (α-crystallin). Interestingly, expanded searches revealed that the gene encoding this protein is found in the genomes of extreme ε-Proteobacteria such as *Nautilia profundicola *and *Caminibacter mediatlanticus*. In addition, *Mycobacterium tuberculosis *has been shown to have two members of the Acr family of molecular chaperones. For example, in 1998, Yuan *et al *demonstrated that *acr *transcription and Acr expression were strongly induced by hypoxic conditions and *in vitro *infection of macrophages, respectively [28]. These authors hypothesized that this protein was important for long term viability and replication during initial *M. tuberculosis *infections. More recently, Wilkinson *et al *determined that two Acr proteins, Acr1 and Acr2, were present in *M. tuberculosis *and were important for the bacterium's interaction with the host [29]. Acr1 has been shown to be induced by exposure to hypoxia or nitric oxide and is associated with bacterial persistence in a non-replicating state, while Acr2 is induced by heat shock, oxidative stress, and uptake by macrophages. In 2005, Stewart *et al *demonstrated that both Acr proteins contribute to persistent infection with *M. tuberculosis*, and suggested that manipulation of *acr *expression can influence the host response to infection [30]. More recently, Stewart *et al *showed that deletion of the *acr *gene resulted in infection of C57BL6 mice with bacillary loads 1-2 log units higher than the wild-type strain [31]. In addition, mice infected with the mutant strain showed high levels of TNF-α, IFN-γ and G-CSF in their serum, suggesting that Acr may play a role in modulating the host response to infection [31]. The presence of this protein in UNSWCD and not in BAA-1457 or any other Campylobacterales further supports the invasive nature of this *C. concisus *strain within the host.

### Flagellin glycosylation in Campylobacter concisus

Like many Gram-negative bacteria with polar flagella, *Campylobacter *flagellins are known to undergo glycosylation. Although pathway variations and sugar modifications exist among strains, most *C. jejuni *strains contain genes for the synthesis of two sugars that link to flagellin: pseudaminic acid (PA) and an acetamidino form of legionaminic acid (LA) [32-34]. The entire PA biosynthetic pathway has been characterized in *Helicobacter pylori *and has been shown to involve six enzymes PseB, PseC, PseH, PseG, PseI and PseF [35]. More recently, the LA biosynthetic pathway was characterized in *C. jejuni *and has also been shown to involve six enzymes, namely LegB, LegC, LegH, LegG, LegI and LegF [36].

**Figure 3:**
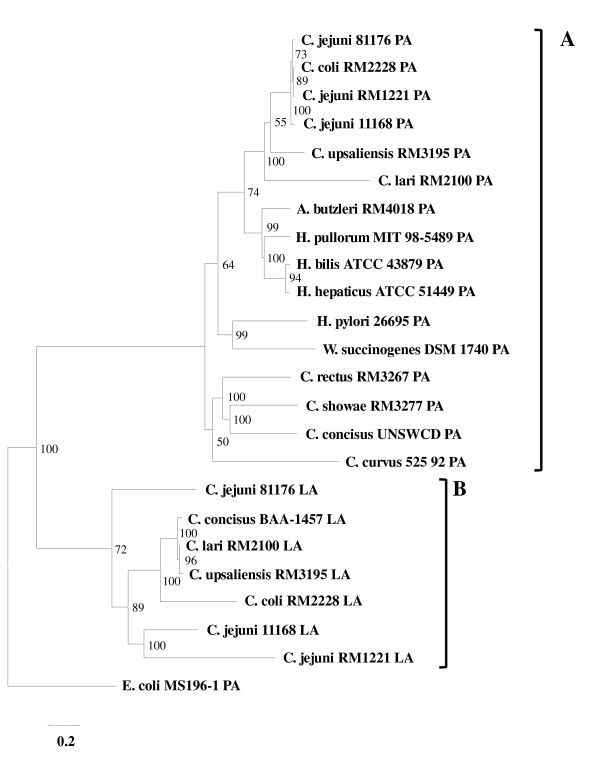
**Phylogenetic analysis of the pseudaminic acid and legionaminic acid biosynthetic pathways of Campylobacterales**. Trees were constructed at the Mobyle@Pasteur portal using the default settings. Trees were visualized using the TreeView software. 0.2 = base substitutions per site.

Bidirectional homology searches against the genomes of UNSWCD and BAA-1457 revealed that UNSWCD contained the PA biosynthetic pathway whereas BAA-1457 contained the LA biosynthetic pathway. To further confirm these findings, a phylogenetic analysis was performed on the six proteins of each pathway for several Campylobacterales (Figure 3). There was a distinct division between the two types of pathways, with the PA and LA pathways clustering into separate groups. Importantly, the six proteins from UNSWCD clustered with the PA pathways whereas the six proteins from BAA-1457 clustered with the LA pathways, confirming the homology searches. *C. concisus *BAA-1457 was the only member of the Campylobacterales to express only a LA biosynthetic pathway. Interestingly, the PA biosynthetic pathways of *Campylobacter *species that contained both pathways within their genome clustered together, revealing an evolutionary closeness within the flagellin glycosylation pathways of these bacteria.

The presence of these pathways were further verified experimentally, where the presence of *pseB *in UNSWCD (Figure 2B) and *legB *in BAA-1457 (Figure 2C) was confirmed by PCR. Six additional *C. concisus *strains (Table 1) were also analyzed, and it was found that they all contained the PA biosynthetic pathway (Figure 2B, C). While currently the function of flagellin glycosylation in *Campylobacter *spp. is not fully understood, perturbations in flagellin glycosylation systems of *Campylobacter *spp. and *H. pylori *have been shown to result in negative effects on filament assembly, autoagglutination, microcolony formation on intestinal epithelial cells, cyto-adherence and invasion [32]. Thus, it is possible that differences in the flagellin glycosylation pathways of *C. concisus *strains may be involved in the variations seen in their adherence and invasive potential. In addition, this provided further evidence that *C. concisus *BAA-1457 was genetically divergent from the other *C. concisus *strains studied. Their similar genetic backgrounds and the presence of only a PA biosynthetic pathway in the seven *C. concisus *strains and only a LA biosynthetic pathway in BAA-1457 makes these bacteria ideal organisms to study these flagellin glycosylation pathways in more detail.

### Protein profiles of Campylobacter concisus

**Figure 4:**
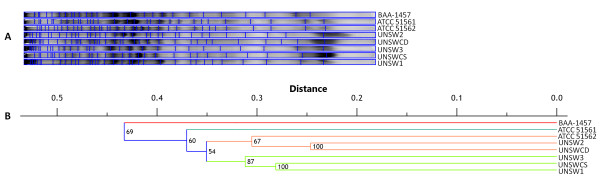
**Protein profiles of eight *Campylobacter concisus *strains**. **(A) **Phoretix 1D pro was employed to cluster the lanes based on banding patterns. (**B**) Dendrogram is a Unweighted Pair Group Method with Arithmetic Mean (UPGMA) Dice coefficient distance tree.

The genomic variations between UNSWCD and BAA-1457 led us to investigate the protein profiles of these two strains and a further six *C. concisus *strains (Table 1) isolated from different disease states. The protein profiles of the total cell lysates of the eight strains were determined using SDS-PAGE, and analyzed using the Phoretix 1D pro software, which clustered the strains based on their banding patterns (Figure 4A, B). The eight strains did not cluster according to the disease state from which they were isolated. This is in line with the findings of Engberg *et al *who reported that strain-specific differences in the ability of *C. concisus *to induce cytolethal distending toxin-like effects on monkey kidney cells had no specific association with disease outcome [4]. Moreover, disease state was not a factor in the hemolytic phospholipase A_2 _activity of *C. concisus *strains isolated from children with diarrhea [37].

At a distance of 0.4 two clades were present, one containing BAA-1457 and the other all other seven strains (Figure 4B). The average similarity of BAA-1457 with the other seven strains was 56.8%, while the mean average similarity of the other seven strains was 64.7% with a standard deviation of 1.7% (Table 2). This indicated that BAA-1457 is highly divergent from the other *C. concisus *strains, providing additional evidence that it is atypical in nature. Interestingly, at a distance of 0.35, three clades were present, one containing BAA-1457, one containing ATCC 51561 isolated from a healthy control and the third containing the other six strains. Excluding the results of BAA-1457, the average similarity of ATCC 51561 with the six other strains was 63.2%, while the average similarity of the six other strains ranged from 64.7-69.0% (mean average similarity of the six other strains = 66.5 ± 1.7%) (Table 2), indicating that the healthy control strain was divergent from the other strains isolated from acute and chronic gastroenteritis and CD. Finally, at a distance of 0.325 four clades were present, where the previous third cluster comprising six strains was split into two groups of three strains (group 1: UNSWCD, UNSW2 and ATCC 51562; group 2: UNSW3, UNSW1 and UNSWCS). While this division was not based on disease state, it may shed light on the discrepancies in the results of previous studies differentiating *C. concisus *strains. That is, techniques with the capacity of differentiating up to two *C. concisus *genomospecies may be observing what is seen at a distance of 0.4, whereas those differentiating up to four molecular groups may be observing that seen at a distance of 0.325.

**Table 2:** Percentage average similarities of the protein profiles of eight *Campylobacter concisus *strains as calculated by Phoretix 1D pro.

	Average similarity (%)							
**Strain**	**UNSWCD**	**UNSW2**	**UNSW3**	**UNSW1**	**BAA-1457**	**UNSWCS**	**ATCC 51562**	**ATCC 51561**

UNSWCD	100	75	62	72	49	71	73	61
UNSW2	75	100	73	62	57	59	66	68
UNSW3	62	73	100	70	62	68	55	60
UNSW1	72	62	70	100	57	72	69	62
BAA-1457	49	57	62	57	100	68	45	60
UNSWCS	71	59	68	72	68	100	61	62
ATCC 51562	73	66	55	69	45	61	100	66
ATCC 51561	61	68	60	62	60	62	66	100

Mean ± SD*	66.1 ± 9.3	65.7 ± 6.8	64.3 ± 6.3	66.3 ± 5.9	56.8 ± 7.8	65.9 ± 5.1	62.1 ± 9.5	62.7 ± 3.1

Error on the mean is the standard deviation (SD).

*Mean of percentage similarities ± standard deviation excludes the 100% value

### Possible markers for the identification of Campylobacter concisus

Due to the large variability observed between *C. concisus *strains UNSWCD and BAA-1457, proteins found in both strains but not in another bacterial species may be ideal markers for the detection of this species. Searches for homologues of the 1593 proteins found to be common between the two strains were conducted against all available bacterial genomes, and 18 proteins were found to be unique to *C. concisus*, of which six were bioinformatically predicted to be secreted (Table 3). These were made up of mostly short (~40-50 amino acids) hypothetical proteins. Of interest was CCC13826_2290 that was 160 amino acids in length and predicted to contain a signal peptide (Table 3). Given that secreted or membrane proteins are more likely to illicit a host immune response, these identified proteins make good targets for species-specific detection through methods such as ELISA. In addition, the low number of proteins unique to these two strains further emphasizes the extent of variability between them.

**Table 3:** Proteins specific to *Campylobacter concisus*.

BAA-1457 ORF	Protein name	Protein length (aa)	Secreted
CCC13826_0031	Hypothetical protein	64	No
CCC13826_0210	Hypothetical protein	56	No
CCC13826_0311	Hypothetical protein	42	Yes (SecP* = 0.61)
CCC13826_0334	Aspartokinase	52	Yes (SecP* = 0.57)
CCC13826_0382	Outer membrane protein	44	No
CCC13826_0423	Transmembrane transport protein	41	Yes (SigP^# ^= 0.98)
CCC13826_0586	Signal transduction sensor protein	38	No
CCC13826_0742	Hypothetical protein	40	No
CCC13826_0909	Hypothetical protein	40	Yes (SecP* = 0.92)
CCC13826_1038	Hypothetical protein	41	No
CCC13826_1698	L-cystine binding protein TcyA	179	No
CCC13826_1719	Hypothetical protein	52	No
CCC13826_1918	Hypothetical protein	44	Yes (SecP* = 0.70)
CCC13826_2161	Ferric uptake regulation protein	47	No
CCC13826_2242	Periplasmic protein	41	No
CCC13826_2291	Hypothetical protein	38	No
CCC13826_2290	Hypothetical protein	160	Yes (SigP^# ^= 1.0)
CCC13826_2294	Hypothetical protein	45	No

Proteins common between *C. concisus *UNSWCD and BAA-1457 with no homologues in other bacterial species were identified through blastp searches. Proteins were predicted to be secreted through the SignalP and SecretomeP servers.

* SecP = Secretome P prediction

# SigP = SignalP prediction

Validation of the presence of the six genes encoding putative taxon-specific markers bioinformatically predicted to be secreted (Table 3) in the eight *C. concisus *strains was performed using PCR and sequencing. Two of the six genes (*ccc13826_0334 *and *ccc13826_2290*) were present within all the eight *C. concisus *strains, thus, providing evidence that they may be good taxon-specific markers for this bacterium. Further investigation, within more *C. concisus *strains, of the potential of these genes to be taxon-specific markers is required.

## Conclusions

This study has elucidated several features that may be responsible for the heterogeneity that exists among *C. concisus *strains, and has identified factors that may explain the differences in their pathogenic potential. The identification of different virulence factors (α-crystallin and zonula occludens toxin) within the two *C. concisus *strains (UNSWCD and BAA-1457) as well as the different types of flagellin glycosylation biosynthetic pathways (pseudaminic acid and legionaminic acid) present interesting avenues to further investigate the differences in pathogenesis of *C. concisus *strains. Importantly, based on the current study, it would appear that BAA-1457 is atypical to other *C. concisus *strains, and thus, as a reference strain for sequence-based analyses on this species, UNSWCD may represent a better candidate.

## Methods

### Comparative bioinformatic analyses

Homology searches were performed using the blastp and blastn tools through the National Center for Biotechnology Information (NCBI) website available at (http://www.ncbi.nlm.nih.gov/) using the default settings. Comparative bioinformatic analyses on the genomes of *C. concisus *strains UNSWCD and BAA-1457 were performed using the Blast2GO tool [38] available at (http://www.blast2go.org/start_blast2go). The Kyoto Encyclopedia of Genes and Genomes (KEGG) [39] available at (http://www.genome.jp/kegg) was employed to determine the biochemical pathways to which genes were assigned. Circular genome visualization was performed using the CGView web-server [40]. Phylogenetic trees on the glycosylation pathways within Campylobacterales were performed at the Mobyle@Pasteur portal available at (http://mobyle.pasteur.fr/cgi-bin/portal.py#welcome) using the default settings. Trees were visualized using the TreeView software available at (http://taxonomy.zoology.gla.ac.uk/rod/treeview.html). The presence and location of signal peptide cleavage sites in the amino acid sequences were predicted using the default settings for Gram-negative bacteria on the SignalP Server 3.0 (http://www.cbs.dtu.dk/services/SignalP/). Non-classically secreted proteins were predicted using the SecretomeP 2.0 Server (http://www.cbs.dtu.dk/services/SecretomeP/).

### Bacterial cultures

*Campylobacter concisus *strains UNSWCD [11], UNSW1, UNSW2, UNSW3 [Kaakoush *et al*, unpublished data], ATCC 51561 [13], ATCC 51562 [13], UNSWCS [14] and BAA-1457 [41] (Table 1) were grown on Horse Blood agar (HBA) supplemented with 6% defibrinated horse blood. Cultures were incubated at 37°C under microaerobic conditions generated using *Campylobacter *Gas Generating Kits BR0056A (Oxoid). The purity of bacterial cultures was confirmed by motility and morphology observed under phase contrast microscopy. Strains were selected based on the diagnosis of the patient they were isolated from because of the observed differences in the pathogenic potential of strains isolated from chronic intestinal diseases and acute intestinal diseases ([14], Kaakoush *et al*, unpublished data).

### Extraction of genomic DNA, polymerase chain reactions and sequencing

DNA extraction was performed using the Puregene Core Kit A (Qiagen) according to the manufacturer's instructions. The concentration and quality of DNA was measured using a Nanodrop ND-1000 Spectophotometer (Nanodrop Technologies). PCR reactions were performed on the DNA extracted from the *C. concisus *strains. The Zot PCRs were performed using the primer pair Zot1 (GCAACTTAGAAAAAGTATCGG) and Zot2 (TAATAGTTCTCGATGAAGCC), which amplify a 979 bp region, and the primer pair ZotF (CTAGAATCAGTTTGTGGAGAT) and Zot2, which amplify a 790 bp region. The *pseB *PCR was performed using the primer pair *pseB*-F (CTAGTTATCTACTCACGCGAC) and *pseB*-R (GGATAGACGGACTAATAACG), which amplifies a 754 bp region. The *legB *PCR was performed using the primer pair *legB*-F (TAGTTATAGGTGCAGCAGGCT) and *legB*-R (GCATCATCTGCACTATATGGT), which amplifies a 817 bp region. The thermal cycling conditions for all three reactions were: 94°C for 5 min, 30 cycles of 94°C for 20 s, 53°C for 20 s, and 72°C for 1 min, followed by 72°C for 5 min.

To validate possible taxon-specific markers six primer pairs targeting the genes *ccc13826_0311, ccc13826_0334, ccc13826_0423, ccc13826_0909, ccc13826_1918 *and *ccc13826_2290 *were designed. The *ccc13826_0311 *PCR was performed using the primer pair 0311F (GAGGTTAGAAATAACAACAGC) and 0311R (TTGCTTGTTCTCATCGTCGTA), which amplify a 226 bp region. The *ccc13826_0334 *PCR was performed using the primer pair 0334F (GAGTGATGATTTTGACTACG) and 0334R (CGAGTCGTAACTATCCTCATC), which amplify a 151 bp region. The *ccc13826_0423 *PCR was performed using the primer pair 0423F (GAAGCATCTAAGCCTGACAGA) and 0423R (ACTCTGCAAACTGACACCAAG), which amplify a 240 bp region. The *ccc13826_0909 *PCR was performed using the primer pair 0909F (GTTAACATAGCTTTGGCAAGG) and 0909R (GCTGTTTTGCGTAAATTTGTG), which amplify a 98 bp region. The *ccc13826_1918 *PCR was performed using the primer pair 1918F (GTGCTTAACAAAAGATGTGGC) and 1918R (GCTGTTTTGCGTAAATTTGTG), which amplify a 114 bp region. The *ccc13826_2290 *PCR was performed using the primer pair 2290F (GAGAGTTTTAGATTTGATGG) and 2290R (CTCATACGAAAGCATCAAGAC), which amplify a 309 bp region. The thermal cycling conditions for all six reactions were: 94°C for 5 min, 30 cycles of 94°C for 20 s, 50°C for 20 s, and 72°C for 20 s, followed by 72°C for 5 min.

To confirm the identities of the products, they were first purified using the QIAquick^® ^PCR Purification Kit (Qiagen) according to manufacturer's instructions. Subsequently, sequencing of the positive PCR products was undertaken using the BigDye™ terminator chemistry (Applied Biosystems). Sequences were resolved on an Automated DNA Sequence Analyser ABI3730 (Applied Biosystems) at the sequencing facility of the School of Biotechnology and Biomolecular Sciences, The University of New South Wales, Sydney, Australia. Products were confirmed using blastn searches available through NCBI.

### SDS-PAGE

*Campylobacter concisus *strains were grown on HBA plates, and bacteria were washed twice in phosphate buffered saline. Following the final wash, packed cells were resuspended in 1 ml TSU buffer (50 mM Tris pH 8.0, 0.1% SDS, 2.5 M urea) and lysed by two freeze-thaw cycles in liquid nitrogen. Lysate proteins (20 μg) were resuspended in 1:1 SDS-PAGE sample buffer (0.375 M Tris pH 6.8, 0.01% SDS, 20% glycerol, 40 mg ml^-1 ^SDS, 31 mg ml^-1 ^DTT, 1 μg ml^-1 ^bromophenol blue). For electrophoretic analyses proteins were further denatured by heating at 95°C for 5 min. Proteins were separated on 12% SDS-PAGE gels by electrophoresis for 1.5 h at 100 V. Gels were stained using Coomassie Brilliant Blue G-250 (Bio-Rad). Phoretix 1D pro software (TotalLab Ltd.; Newcastle Upon Tyne, NE, UK) was employed to determine the percentage similarities between the protein profiles of the *C. concisus *strains, and to cluster the lanes based on banding patterns.

## Competing interests

The authors declare that they have no competing interests.

## Authors' contributions

NOK participated in the study design, carried out the genetic and proteomic studies, participated in the bioinformatic studies and drafted the manuscript. NPD participated in the study design, carried out the bioinformatic studies and helped to draft the manuscript. MRW participated in the study design and bioinformatic studies and helped to draft the manuscript. MJR participated in the proteomics studies. KJ participated in the genetic studies. HM conceived the study, and participated in its design and helped to draft the manuscript. All authors read and approved the final manuscript.

## Supplementary Material

Additional file 1 **Table S1. Strain-specific proteins in *Campylobacter concisus *BAA-1457**. Proteins of *C. concisus *BAA-1457 with no homologues in UNSWCD were identified through blastp searches. Proteins were determined to be expressed through Orbitrap mass spectrometry analyses [18]. Click here for file

Additional file 2 **Table S2. Strain-specific proteins in *Campylobacter concisus *UNSWCD**. Proteins of *C. concisus *UNSWCD with no homologues in BAA-1457 were identified through blastp searches. Proteins were determined to be expressed through Orbitrap mass spectrometry analyses [18]. Click here for file
